# Mygalin: An Acylpolyamine With Bactericidal Activity

**DOI:** 10.3389/fmicb.2019.02928

**Published:** 2020-01-10

**Authors:** Abraham Espinoza-Culupú, Elizabeth Mendes, Hector Aguilar Vitorino, Pedro Ismael da Silva, Monamaris Marques Borges

**Affiliations:** ^1^Ph.D. Program in Biotechnology, University of São Paulo, São Paulo, Brazil; ^2^Bacteriology Laboratory, Butantan Institute, São Paulo, Brazil; ^3^Institute of Marine and Environmental Technology, University of Maryland Center for Environmental Science, Columbus Center, Baltimore, MD, United States; ^4^Laboratory for Applied Toxinology (LETA), Butantan Institute, São Paulo, Brazil

**Keywords:** acylpolyamine, Mygalin, oxidative stress, *E. coli*, antimicrobial, biomolecule

## Abstract

Inappropriate use of antibiotics favors the selection and spread of resistant bacteria. To reduce the spread of these bacteria, finding new molecules with activity is urgent and necessary. Several polyamine analogs have been constructed and used to control microorganisms and tumor cells. Mygalin is a synthetic acylpolyamine, which are analogs of spermidine, derived from the hemolymph of the spider *Acanthoscurria gomesiana*. The effective activity of polyamines and their analogs has been associated with their structure. The presence of two acyl groups in the Mygalin structure may give this molecule a specific antibacterial activity. The aim of this study was to identify the mechanisms involved in the interaction of Mygalin with *Escherichia coli* to clarify its antimicrobial action. The results indicated that Mygalin exhibits intense dose and time-dependent bactericidal activity. Treatment of *E. coli* with this molecule caused membrane rupture, inhibition of DNA synthesis, DNA damage, and morphological changes. The esterase activity increased along with the intracellular production of reactive oxygen species (ROS) after treatment of the bacteria with Mygalin. In addition, this molecule was able to sequester iron and bind to LPS. We have shown that Mygalin has bactericidal activity with underlying mechanisms involving ROS generation and chelation of iron ions that are necessary for bacterial metabolism, which may contribute to its microbicidal activity. Taken together, our data suggest that Mygalin can be explored as a new alternative drug with antimicrobial potential against Gram-negative bacteria or other infectious agents.

## Introduction

There is a constant need for new antibiotics due to the rapid development of antibiotic resistance. Extensive and prolonged use and inappropriate prescribing of antibiotics favor rapid selection of resistant microorganisms. This resistance is multifactorial but is associated with poor hygiene and often a delay in the diagnosis of bacterial infections. Each year, microorganisms develop sophisticated and complex mechanisms to circumvent the antibiotics in use, resulting in serious and prolonged life-threatening infections (Laxminarayan et al., 2013). The urgency for developing new antibiotics is evident as infectious diseases are one of the leading causes of mortality worldwide, especially in hospital settings (Fair and Tor, 2014; Ventola, 2015). It is estimated that by 2050, approximately 10 million people will die due to antimicrobial resistance (O’Neil, 2014). The pharmaceutical industry has reduced investments in research on new antibiotics due to economic issues since these companies seek an immediate financial return, and this research is carried out over long periods. Diverse natural products in this therapeutic class have been presented as alternatives for meeting new targets, since such products provide novel and diverse chemicals, aiding in the control of microorganisms (Bush, 2004; Brown and Wright, 2016). Natural polyamines are a group of endogenous cationic compounds that differ in the number of amine groups inserted into the molecule. Putrescine is a diamine, while spermidine and spermine contain three and four amino groups, respectively. The differences in these clusters generate different functions between these molecules and are associated with broad biological functions. These molecules can control cell proliferation and differentiation and regulate protein synthesis and gene expression as they can interact with portions of DNA and RNA (Igarashi and Kashiwagi, 2010), promoting conformational changes in the structure and function of these molecules (Panagiotidis et al., 1995). The largest fraction of polyamines present in eukaryotic cells and *Escherichia coli* was found in complexed RNA (Igarashi and Kashiwagi, 2010). Polyamines also modulate intracellular signals (Huang et al., 2005) and immune functions, depending on their nature (Zhang et al., 1997; Haskó et al., 2000). In addition, these molecules have been shown to bind and alter DNA and RNA (Pegg, 2016). In infections by pathogenic microorganisms, polyamines regulate virulence gene expression (Jelsbak et al., 2012), modify bacterial resistance to oxidative stress (Ha et al., 1998; Chattopadhyay et al., 2003), interfere with biofilm formation (Patel et al., 2006) and response to antibiotics depending on the characteristics of the bacterial structure and the antimicrobial agent (Kwon and Lu, 2007). In *E. coli*, these molecules may control membrane permeability by blocking purine channels (Dela Vega and Delcour, 1996), while synthetic polyamine analogs increase membrane permeability by disruption of LPS integrity (Yasuda et al., 2004). However, the molecular mechanisms involved in these events are mostly unknown. Mygalin is a synthetic molecule originally isolated from hemocytes of the spider *Acanthoscurria gomesiana* and is characterized as a bis-acylpolyamine N1, N8-bis (2,5-dihydroxybenzoyl) spermidine of 417 Da (Pereira et al., 2007). This molecule also does not promote cytotoxicity of murine splenocytes and interferes with innate immunity (Mafra et al., 2012). Polyamines play an important role in the pathogenesis and control of some infections (Blanchet et al., 2016) and evidence suggests an association between the structure and microbicidal activity of some polyamine analogs (Balakrishna et al., 2006). Our aim was to analyze the mechanisms involved in the microbicidal activity of Mygalin using *E. coli* as a model to explore the potential of this compound for the development of a new alternative antibiotic.

## Materials and Methods

### Chemicals and Reagents

Antibiotics (ciprofloxacin, gentamicin, and ampicillin), 2,5-dihydroxybenzoic acid (gentisic acid), spermidine, HBTU, EDTA, DNTB, DAPI, propidium iodide (PI), carboxyfluorescein diacetate assay (CFDA), Triton X-100, LPS from *E. coli* serotype:0111:B4 and agarose were purchased from Sigma Chemical Co. (St. Louis, MO, United States), and CM-H2DCFDA was purchased from Thermo Fisher Scientific (Waltham, MA, United States).

### Synthesis of Mygalin

Mygalin was synthesized at the Center for Research on Toxins, Immune-Response and Cell Signaling (CeTICS – CEPID), Laboratory for Applied Toxinology (LETA) – Butantan Institute and provided by Dr. Pedro Ismael da Silva Jr. Mygalin was synthesized according to the classical method of peptide chemistry (Atherton, 1989). Briefly, the synthesis was carried out in HBTU solution (Knorr et al., 1989) for the esterification of the carboxyl group of gentisic acid and thus permitting the formation of one carboxamide by formal condensation of two primary amino groups from spermidine with a carboxylic group of two molecules of gentisic acid (2,5-dihydroxybenzoic acid). Data is available on the Ontology of Chemical Entities of Biological Interest (ChEBI) database as Mygalin (CHEBI:64901) (EBI, 2014).

### Bacterial Strain, Culture Condition and Antibacterial Assay

All tests were performed with *E. coli* DH5α. The bacterial culture was grown at 37°C using 10 mL of Luria-Bertani broth (Kasetty et al., 2011) in a shaker incubator at 180 rpm, and 100 μL of culture grown overnight was reinoculated in 20 mL of Luria-Bertani broth and allowed to grow to the initial log phase OD_620_ of 0.3 (10^8^ cells/mL). Then, 10^5^ cells/mL were diluted 1:10 in 100 μL of M9 (minimal medium). The antimicrobial activity of the drugs was determined in 96-well microplates containing serial dilutions of Mygalin. The plates were then incubated in a shaker incubator at 180 rpm at 37°C and were protected from light, and the minimum inhibitory concentration (MIC) was determined after 18 h. H_2_O_2_ was used as the positive control of the reaction. Plates were prepared in triplicate, and light was excluded during the experiments. To assess the intrinsic property of Mygalin, 10^7^ cells/mL were individually treated with two concentrations of Mygalin and H_2_O_2_ (0.5 and 1 mM), 1 mM spermidine and 2,5-dihydroxybenzoic acid in PBS at 37°C for 18 h (Kumar et al., 2011).

### Cell Viability Assay With Resazurin-Assay and CFU Definition

Bacterial viability was determined by two methods: counting the colony forming units (CFU) and the resazurin test (Riss et al., 2004; Sarker et al., 2007). For the resazurin test, plates containing 10^4^ cells/mL were incubated at 37°C and protected from light for 18 h, and 20 μL of resazurin solution (0.2 mg/mL) was added thereafter. The reaction was incubated for 2 h, and the color change was monitored (blue, indicating non-viable and purple or pink, indicating viable) and measured at 550 and 595 nm.

### Mechanism of Action of Mygalin Against *E. col*i

To study the mechanism of action of Mygalin, we examined the action against DNA, membrane integrity, protein synthesis, ROS generation and ferrous ion-chelating activity.

### DNA Oxidative Damage

Bacterial DNA damage was evaluated by two different methods: using pure DNA from *E. coli* DH5α incubated with the drug (*in vitro* effects) and DNA isolated from drug-treated bacteria (*in vivo* effects). The reaction product was analyzed by alkaline electrophoresis gel (Drouin et al., 1996).

### *In vitro* Assay

For this assay, the DNA was purified using the Wizard^®^ Genomic DNA Purification Kit. To assess the effect of pH on the drug activity against purified bacterial DNA, 1 μg of DNA was incubated for 2 h with Mygalin or spermidine (0.25; 0.5 and 1 mM) in buffers with different pH values (citrate pH 3.6, phosphate, pH 7.2 and bicarbonate-carbonate, pH 10.6). As a positive control of DNA damage, the Fenton reaction was used, where 1 μL of FeSO4 (1 mM), 1 μL of 2% v/v H_2_O_2_, and 3 μL of Milli-Q water were mixed in a total reaction volume of 15 μL (Cheng et al., 2013). In another assay, to rule out the presence of DNases in the samples, Mygalin was treated with DNase inhibitors such as sodium citrate (100 mM) and EDTA (100 mM) (Kolarevic et al., 2014) or subjected to physical damage by heating at 75°C for 15 min and incubate with 1 μg of DNA. To assess the effect of spermidine on the protection of DNA the reaction was incubated for 2 h with spermidine (0.5 and 1 mM) and Mygalin (0.25; 0.5 and 1 mM). The treated DNA was used as a template to amplify the ICD (Isocitrate dehydrogenase) house-keeping gene using the primers F: 5′-ATGGAAAGTAAAGTAGTTGTTCCGGCACA-3′ and R 5′-GGACGCAGCAGGATCTGTT-3′ (Wirth et al., 2006). After treatment, equal amounts of DNA or PCR products were mixed with alkaline charge buffer, loaded into a 1% agarose gel under alkaline conditions at 70 V, and then stained with gelRed^TM^ (Biotinun). All DNA images were obtained with an electronic documentation system (UVITEC, Cambridge).

### *In vivo* Assay

Bacteria in the exponential growth phase (10^6^ CFU/mL) were incubated with Mygalin (0.5 and 1 mM), spermidine (1 mM) or H_2_O_2_ (0.5 and 1 mM) at 37°C for 5 and 18 h. After the incubation, the cultures were centrifuged at 13,000 rpm. The pellet was washed once with PBS, and DNA was isolated with the Wizard^®^ Genomic DNA Purification Kit. Equal amounts of DNA sample were mixed with alkaline buffer as described above.

In another assay, to visualize DNA fragmentation, bacterial cultures treated for 18 h with Mygalin or H_2_O_2_ were permeabilized with ethanol and stained with DAPI. These samples were fixed on a slide with 1% agarose and visualized by confocal microscopy (Kumar et al., 2011).

### Inhibitory Effect of Mygalin on DNA Synthesis as Determined by Filamentation Assay

The inhibitory effect of Mygalin on DNA synthesis was evaluated by the *E. coli* filamentation assay described by Alfred et al. (2013) with a slight modification. Log phase bacteria (10^8^ cells/mL) were cultured at 37°C in M9 medium and treated with Mygalin (0.5 mM) or ciprofloxacin (0.5 mM) for 3 h. A total of 20 μL of the sample was placed on a glass slide, air-dried and stained with Gram staining. The cells were visualized by light microscopy (1000×). All assays were performed in triplicate.

### Action on Membrane Integrity and Esterase Activity in *E. col*i

To evaluate the damage caused by Mygalin to the cell membrane, *E. coli* (10^8^ CFU/mL) were cultured with Mygalin (0.5 mM) or ampicillin (0.5 mM) for 5 h and then washed and suspended in 50 mM phosphate buffer, pH 7.0. Then, the bacteria were incubated with PI at a final concentration of 60 μM and kept in the dark for 15 min (Nocker et al., 2011). Then, 20 μL of sample was added to a slide with 1% agarose, the slide was covered with a coverslip, and the membrane integrity was analyzed by confocal microscopy. For the esterase activity assay, untreated bacteria and those treated with the drugs for 4 h were washed once in PBS and suspended in 50 mM phosphate buffer. A total of 180 μL of bacterial suspension was placed on black COSTAR^®^ 96-well microplates with the addition of 20 μL of CFDA (250 μM). The samples were incubated for 30 min in the dark, and fluorescence was measured at 485/535 nm excitation/emission wavelengths (Nocker et al., 2011; Hyldgaard et al., 2015).

### Determination of Reactive Oxygen Species (ROS)

For this assay, one milliliter of *E. coli* (10^6^ cells/mL) obtained in the exponential growth phase was washed with PBS and resuspended in 1 mL of 50 mM phosphate buffer. The mixtures were treated or not treated with Mygalin (0.25 and 0.5 mM) or H_2_O_2_ (0.25, 0.5, and 1 mM) for 15 min at room temperature. After that, CM-H2DCFDA (1 μM) was added. Subsequently, 100 μL of the bacteria were transferred to black 96-well microplates (Dong et al., 2015), and the fluorescence was measured every 30 min using a PerkinElmer Victor 3^TM^ 1420 Multilabel Counter Fluorometer with 485/535 nm excitation/emission wavelengths.

### Mygalin-LPS Interactions

The LPS from *E. coli* serotype: 0111: B4 was prepared in water endotoxin-free, and 50 μL of LPS solution (10, 20, 40, 80,160, 320, and 640 ng/mL) was incubated with a fixed concentration of Mygalin (500 μM) for 1 h at 37°C. The interaction between Mygalin and LPS was determined by monitoring the change in the absorbance of Mygalin, using 2 μL of each sample in a NanoVue Plus^TM^ spectrophotometer (GE Healthcare Life Science) with a Pathlength of 0.5 mm. The plates containing the samples were prepared in triplicate, and light was excluded during the experiments. The blank was endotoxin-free water (Lakshminarayanan et al., 2016).

### Glutathione (GSH) Levels and Protein Profile

*Escherichia coli* (10^9^ cells/mL) were treated with Mygalin, ciprofloxacin, and gentamicin for 18 h, then centrifuged at 13,000 rpm for 5 min, thoroughly washed three times with PBS. Next, the cells were resuspended in 250 μL of lysis buffer (25 mM Tris–HCl, pH 7.5, 100 mM NaCl, 2.5 mM EDTA, 20 mM NaF, 1 mM Na3VO4, 0.5% Triton X-100 and 1 mM PMSF). The cells were sonicated on ice for five cycles of 20 s at 50 W power and allowed to rest for 1 min on the HD 2070 ultrasonic homogenizer. The cell lysate suspension was centrifuged at 4°C and 14,000 rpm for 10 min. The recovered supernatant was used to quantify the protein level using a Pierce^TM^ BCA Protein Assay Kit (Thermo Scientific, Waltham, MA, United States). To measure glutathione (GSH) levels, Ellman’s reagent was used (Ellman, 1959); 25 μg of protein was added into 96-well microplates containing 50 μL of solution (50 nM GR, 50 mM Tris–HCl (pH 7.5), 200 μM NADPH, 1 mM EDTA, 1 mM DTNB) (Zou et al., 2017). The plates were read after 15 min of incubation at room temperature at 412 nm. The GSH levels were estimated after determination of the protein levels. Cell lysates were placed in SDS gel loading buffer at 90°C for 10 min and then separated with 10% polyacrylamide gel electrophoresis (SDS-PAGE), as described by Laemmli (1970). Equal amounts of protein were loaded per lane. Protein separation was performed at 4°C for 1.5 h at 100 V in a Hoefer miniVE (Amersham Biosciences). Proteins were visualized by Coomassie Blue Staining.

### Ferrous Ion-Chelating Activity

The ferrous ion-chelating activity of Mygalin was investigated according to Baccan et al. (2012) and Vitorino et al. (2015) with ferric nitrilotriacetate. Fe(NTA) was prepared by the titration of 70 mM NTA to pH 7.0 with NaOH, followed by the addition of solid FAS (ferric ammonium sulfate) to attain a final iron concentration of 20 mM, and the solution was heated in a water bath for 1 h. Aliquots of 10 μL of 2 μM Fe(NTA) in HBS/Chelex were transferred to a flat, transparent 96-well microplate and treated with 10 μL of Mygalin (0–1000 μM), followed by 180 μL of a mixture of 50 μM DHR and ascorbic acid (40 μM) in Milli-Q water. The kinetic curve was registered with excitation/emission wavelengths of 485/520 nm at 25°C for 40 min. The slopes (F min^–1^, where F stands for arbitrary fluorescence units), calculated from 15–40 min, were then plotted against the chelator concentration. The experiment was conducted in quadruplicate.

### Statistical Analysis

All results were analyzed using Student’s *t*-test and one-way ANOVA, and the difference between groups was determined by the Tukey–Kramer test or Dunnett’s multiple comparisons analyzed by the GraphPad Prism 7 program (Graph Pad, San Diego, California). The data were considered statistically significant at *p* < 0.05, and the results represent the mean and standard error of the mean (±SEM) from at least three independent experiments.

## Results

### Chemical Structure of Mygalin and Effect of Mygalin on *E. coli* Viability

**FIGURE 1:**
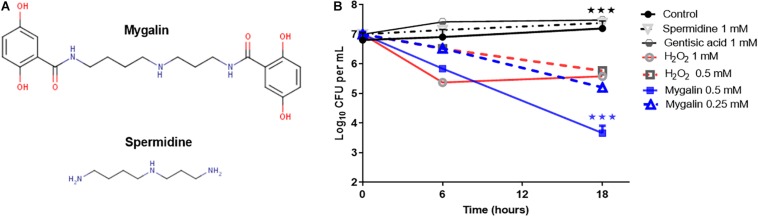
Structure of Mygalin and time-killing curves for *Escherichia coli* DH5α. **(A)** Structural difference between Mygalin and spermidine. **(B)** Bacteria (10^7^ CFU/mL) were grown in M9 medium and diluted to 10^3^ in 100 μL, as described in the section “M&M,” then incubated at 37°C for 6 and 18 h with or without the indicated concentrations of Mygalin (0.5 and 0.25 mM), spermidine (1 mM), gentisic acid (1 mM), and H_2_O_2_ (0.5 and 1 mM). Viable cell counts were determined by measuring the number of colony forming units (CFU) after 24 h. Data represent ±SEM of three independent experiments (^∗∗∗^*p* < 0.001).

To explore the microbicidal activity of Mygalin, the *E. coli* DH5α model was used. Figure 1A shows the chemical structure of Mygalin (Pereira et al., 2007) compared with spermidine including the presence of two acyl groups, which can be used to differentiate between these molecules. To evaluate the effect of Mygalin on *E. coli* viability (Figure 1B), a fixed number of bacteria were treated for 6 and 18 h, and the number of colonies formed (CFU) was counted on LB agar plates after 6 and 24 h of incubation. Bacterial treatment for 6 h reduced the bacterial viability (CFU) compared to the control containing spermidine and gentisic acid, both used for Mygalin synthesis. The reduction was more apparent over 18 h of treatment with 0.5 mM Mygalin treatment showing greater effects than 1 mM H_2_O_2_, which was used as a positive control. The specificity of the reaction was confirmed since the treatment with spermidine or gentisic acid had no effect on the viability of the bacteria, with CFU values similar to the control without treatment.

### Interaction of Mygalin With DNA

#### *In vitro* Model

**FIGURE 2:**
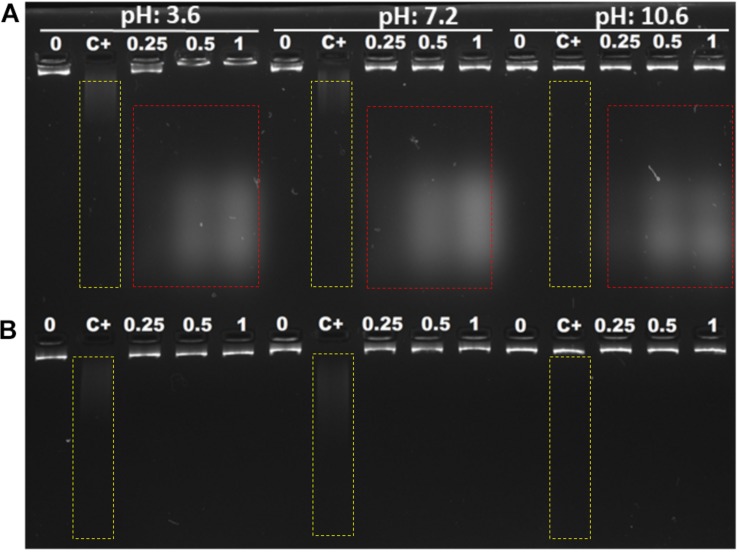
Interaction of *E. coli* genomic DNA with Mygalin and spermidine. A total of 1 μg of DNA was incubated with different amounts of Mygalin **(A)** and spermidine **(B)** (0.25, 0.5, and 1 mM) in three buffers: citrate buffer, pH: 3.6; phosphate buffer, pH: 7.2; and bicarbonate-carbonate buffer, pH: 10.6 at 37°C for 2 h. The reaction mixtures were applied to 1% alkaline agarose gel and stained with gelRed^TM^. The positive control of DNA damage was FeSO4 + H_2_O_2_, the integrity of DNA was analyzed in agarose gels, and the images were acquired using an electronic documentation system (UVITEC, Cambridge). The figure is representative of two independent experiments with similar results.

**FIGURE 3:**
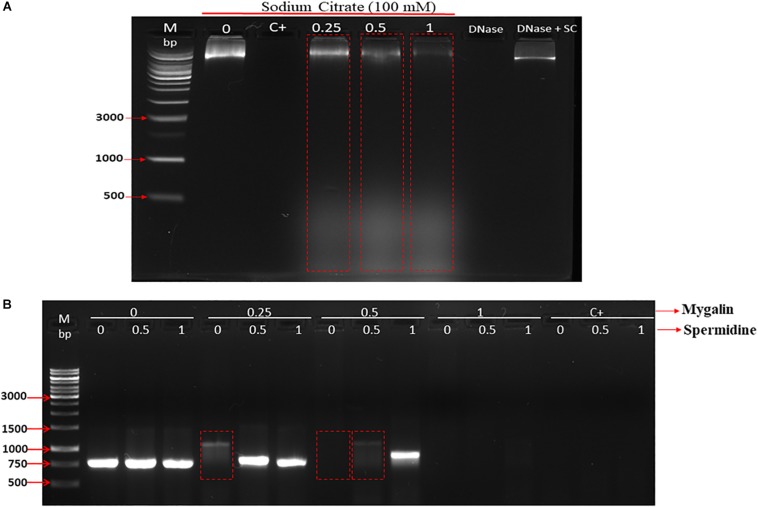
Effect of addition of DNase inhibitor and spermidine protection on oxidative damage: **(A)** Gel electrophoresis, of DNA treated with Mygalin (0.25, 0.5, and 1 mM) in the presence of the DNase inhibitor (sodium citrate 100 mM). Controls: untreated DNA (0), Fenton reaction (C+), sodium citrate (SC). **(B)** PCR of Mygalin-treated DNA (0.25, 0.5, and 1 mM) plus spermidine (0.5 and 1 mM). Controls: untreated DNA (0), Fenton reaction (C+), Ladder Gene Ruler 1 Kb (M).

Polyamines bind to nucleic acids, causing their condensation (Feuerstein et al., 1991). Since Mygalin is an acylpolyamine analog of spermidine, this led us to study DNA as its first target. Initially, we evaluated whether pH influences the action of Mygalin against bacterial DNA. Three buffers with different pH values (3.6, 7, and 10.6) were used to dilute the Mygalin. We confirmed, using alkaline electrophoresis gel, that the effect of Mygalin (0.25–1 mM) includes oxidative DNA damage (Figure 2A). This effect occurred in a variable pH range from acidic to alkaline, as highlighted in the figure (red box). The same assay performed with spermidine (Figure 2B) did not cause any DNA damage. The Fenton reaction was used as a positive control of DNA damage (yellow box). We observed that this reaction was neutralized, differing from that observed with Mygalin at the alkaline pH (Figures 2A,B). Another assay was performed to rule out the presence of external DNase (Figure 3A), it was shown that the addition of the DNase inhibitor did not alter the DNA damage caused by Mygalin, unlike the DNA samples treated with DNases alone. This result indicates that the breakdown of DNA was caused by treatment with Mygalin. Figure 3B, shows that the addition of spermidine (0.5 and 1 mM) protected DNA samples treated with Mygalin at doses below 0.5 mM. However, this effect was not observed with Mygalin (1 mM) or the Fenton reaction (positive control), confirming that high concentrations of Mygalin causes irreversible damage.

#### *In vivo* Model

**FIGURE 4:**
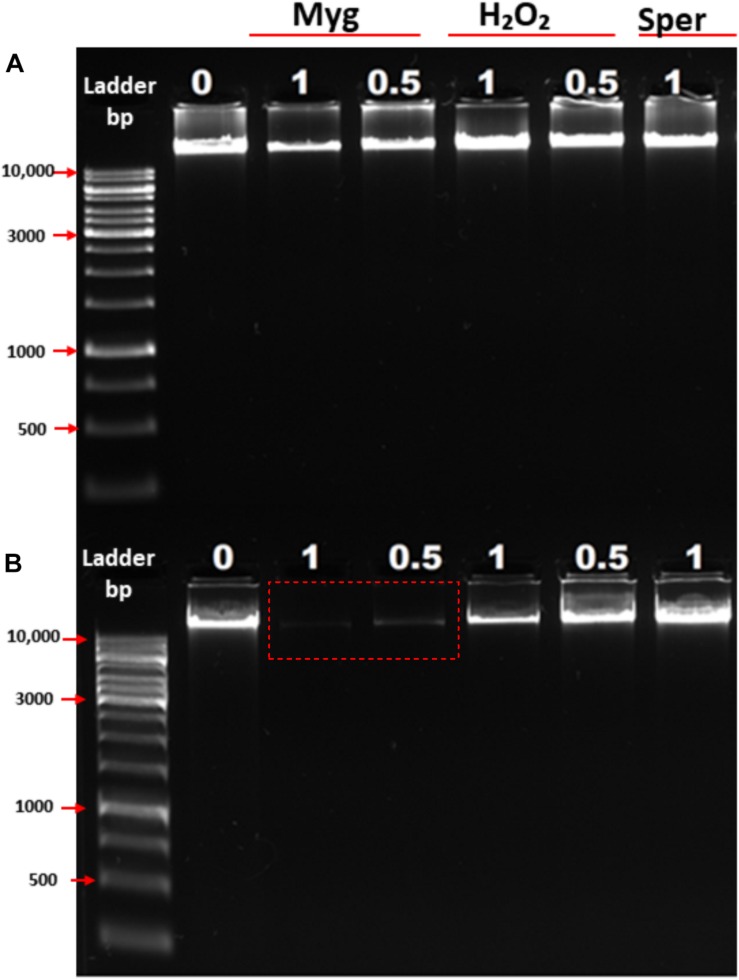
Effect of Mygalin, hydrogen peroxide and spermidine on the integrity of *E. coli* DNA. Bacteria (10^6^ CFU/mL) in the log phase were treated with or without 0.5 and 1 mM of Mygalin, H_2_O_2_ or spermidine (1 mM) for 5 **(A)** and 18 h **(B)** at 37°C. DNA was isolated using the Wizard^®^ Genomic DNA Purification Kit. DNA samples (1 μL) from each group were analyzed in 1% alkaline agarose gel and stained with gelRed^TM^. Myg = Mygalin, Sper = spermidine, H_2_O_2_ = hydrogen peroxide. The figure is representative of three independent experiments with similar results.

To further examine the ability of Mygalin to promote DNA damage, we used viable bacteria obtained in the exponential phase of growth to confirm the effect of previous *in vitro* assays. Bacteria were cultured with Mygalin (0.5 and 1 mM) for 5 and 18 h and later washed with PBS, and the pellet was used to extract the genomic DNA. The DNA was analyzed by electrophoresis under alkaline conditions (Figure 4). After 5 h incubation with Mygalin, there was a marked reduction in genomic DNA, which accentuated at 18 h, when DNA was no longer visualized. In bacteria treated with the same concentration of H_2_O_2_, the effect was less pronounced, while spermidine did not cause any DNA damage. This confirms the previous data from *in vitro* assays, showing that the treatment of *E. coli* with Mygalin promotes DNA damage differing from that observed with spermidine.

### DNA Labeling After Treatment With Mygalin

**FIGURE 5:**
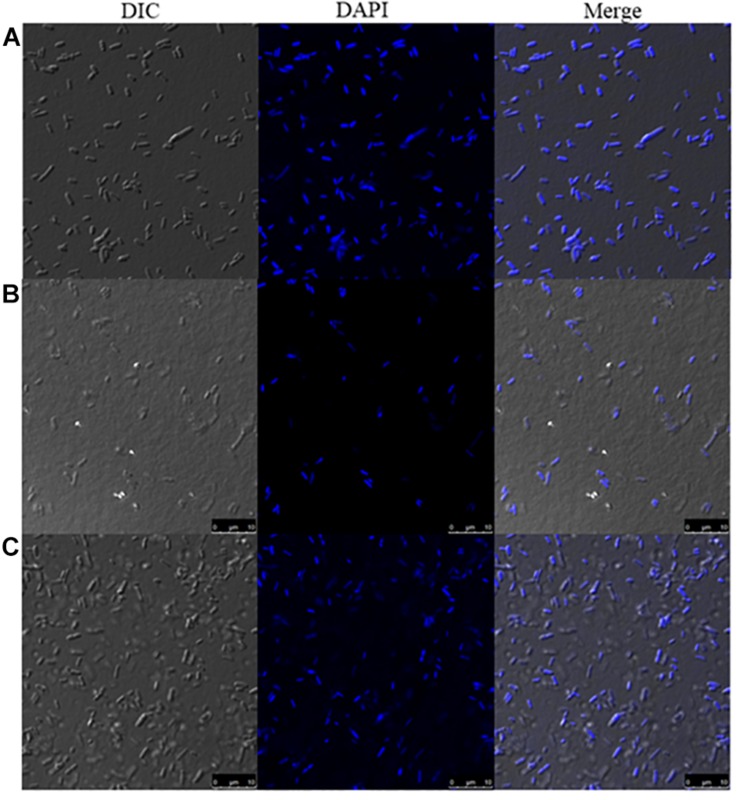
Confocal microscopy of Mygalin-treated *E. coli* stained with DAPI or subjected to DNA fragmentation. Bacteria (10^6^ CFU/mL) untreated **(A)** or treated for 18 h with **(B)** Mygalin (0.5 mM) or **(C)** H_2_O_2_ (0.5 mM) as a control were permeabilized with ethanol and stained with DAPI (3 μM). Confocal microscopy was used to visualize DNA fragmentation. DIC = Differential Interference Contrast.

DAPI is a fluorescent dye that selectively binds DNA to form a strong fluorescent DNA-DAPI complex with high specificity. When DAPI is intercalated into cellular DNA, it fluoresces and DAPI has been widely used to evaluate DNA structural damage. This approach was used to confirm the bacterial DNA damaging effects of Mygalin. In this assay, bacteria treated with or without Mygalin (0.5 mM) or H_2_O_2_ as a positive control were permeabilized and stained with DAPI and analyzed by confocal microscopy. Figure 5 shows that untreated bacteria (Figure 5A) were completely stained, demonstrating the integrity of the DNA. However, those treated with either Mygalin (Figure 5B) or H_2_O_2_ (Figure 5C) did not show extensive staining due to DAPI not being intercalated with the double strand of the damaged DNA. These data reinforce the idea that Mygalin causes oxidative damage in bacterial DNA.

### Inhibition of DNA Synthesis *in vivo* by Mygalin Using *E. coli* Filamentation Assay

**FIGURE 6:**
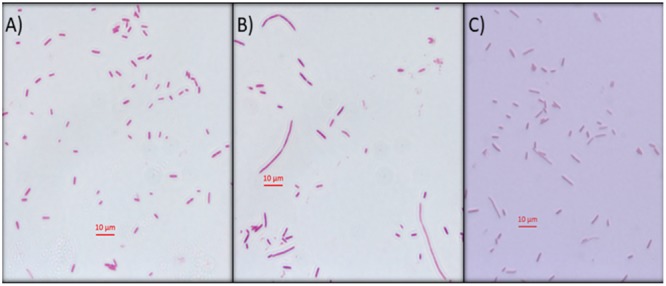
*Escherichia coli* filamentation assay as indicative of DNA inhibition by Mygalin. **(A)** Untreated *E. coli* (10^6^ CFU/mL) or **(B)** *E. coli* (10^6^ CFU/mL) treated for 3 h with 0.5 mM of Mygalin **(B)** or ciprofloxacin **(C)** were stained by Gram stain. Light microscopy (magnification 1000×) was used for cell division and filament formation analysis. Scale 10 μm.

Another approach used to determine the interference of this compound on *E. coli* DNA was inhibition of DNA synthesis using the filamentation assay (Alfred et al., 2013). To test if Mygalin uses this system, the bacteria were treated for 3 h with 0.5 mM of Mygalin (Figure 6B) or ciprofloxacin (Figure 6C) as a positive control and their morphology and filament formation were compared with those of untreated cells (Figure 6A) by light microscopy (Figure 6). The results showed that treatment with Mygalin or ciprofloxacin interfered with cell division, resulting in long bacterial filaments when compared to untreated cells. These results indicate that Mygalin and ciprofloxacin can bind to DNA, inhibiting DNA synthesis *in vivo*.

### Action of Mygalin on Cell Membrane and Esterase Activity

**FIGURE 7:**
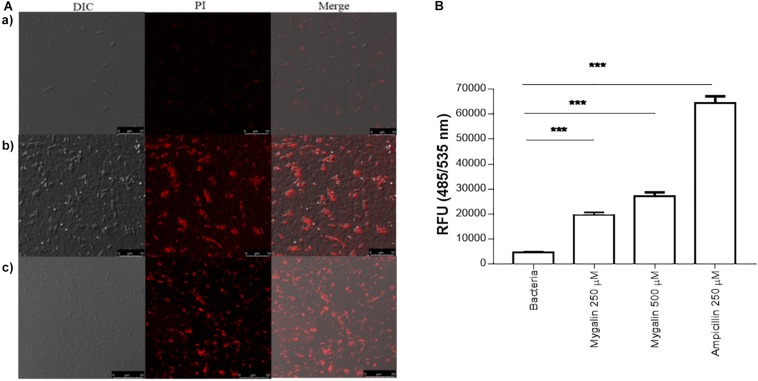
Effect of Mygalin on membrane integrity measured by propidium iodide uptake and esterase activity of *E. coli*. **(A)** Propidium iodide uptake. **(a)** Untreated *E. coli* (10^8^ CFU/mL) or *E. coli* treated with 0.5 mM Mygalin or **(b)** 0.5 mM ampicillin **(c)** as a positive control were incubated for 5 h at 37°C, fixed in agarose and observed by confocal microscopy. Bacteria in red are indicative of dead or membrane-damaged cells. **(B)** Effect of Mygalin on the esterase activity of *E. coli*. Bacteria (10^8^ CFU/mL) were treated with Mygalin (250 and 500 μM) or ampicillin (250 μM) for 4 h and stained with CFDA. The fluorescence intensity was read at 485/535 nm. The results represent the fluorescence intensity and are reported as relative fluorescence units (RFU). Data represent ±SEM of three independent experiments (^∗∗∗^*p* < 0.001).

In addition to DNA damage, antimicrobial drugs can act on several other mechanisms, including disrupting the cell membrane (Epand et al., 2016). To test the effect of Mygalin on the *E. coli* membrane, bacteria were cultured for 5 h in the absence (a) or presence of 0.5 mM of Mygalin (b) or 0.5 mM of ampicillin (c) and a PI uptake assay was used to measure membrane permeability changes under confocal microscopy (Figure 7A). The results indicate that untreated bacteria were impermeable to PI, while *E. coli* treated with Mygalin or ampicillin showed PI uptake, visualized as cells with red staining. This suggests that Mygalin can promote cell membrane rupture in *E. coli.* To monitor this membrane change, the intracellular esterase activity was assayed using the CFDA. If the membrane breaks, fluorochrome diffuses into the cells being cleaved, and a highly fluorescent product is released that corresponds to esterase activity (Hong et al., 2015). The treatment of *E. coli* for 4 h with 0.25 and 0.5 mM of Mygalin increased the dose-dependent esterase level, which was four times higher than that of the untreated control, while ampicillin (0.25 mM) increased the activity more than ten-fold. These results confirm that Mygalin damages the bacterial cell membrane (Figure 7B).

### Effect of Mygalin on the Glutathione Level and Protein Profile in *E. col*i

**FIGURE 8:**
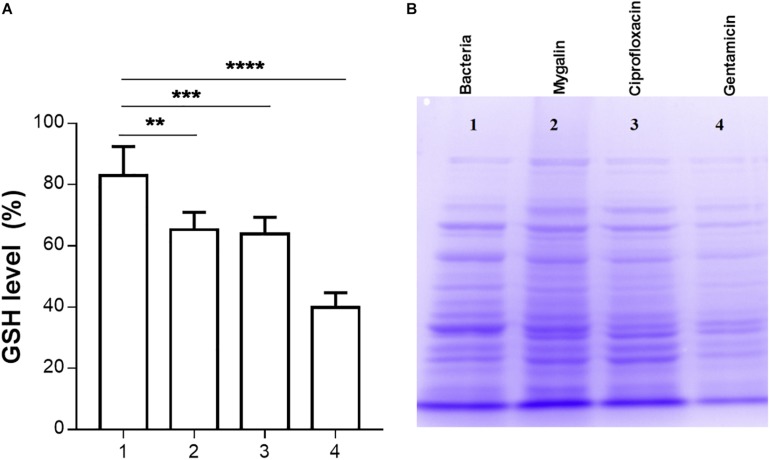
Effect of Mygalin on intracellular GSH levels and protein profile. Bacteria (10^9^ CFU/mL) were treated or not treated for 18 h, sonicated and centrifuged and the supernatant was used to measure intracellular GSH and perform protein profile analysis. **(A)** GSH was determined in untreated *E. coli* lysate (1) or that treated with 0.5 mM Mygalin (2), ciprofloxacin (3) or gentamicin (4). GSH levels were defined after the comparison between the control and treated groups using the DTNB reduction assay. **(B)** Electrophoretic protein profile (SDS-PAGE) from the *E. coli* lysate supernatant exposed to the treatments mentioned above. Proteins were visualized by Coomassie brilliant blue staining. Data represent ±SEM of three independent experiments. Statistical significance using Dunnett’s multiple comparisons test comparing untreated and treated groups. ^∗∗^*p* < 0.01 and ^∗∗∗^*p* < 0.001.

Glutathione has several functions, including a role in the metabolism of peroxides, inactivation of free radicals, and maintenance of oxidation-reduction potential, in addition to participating in reactions involving the synthesis of proteins (Smirnova and Oktyabrsky, 2005). We evaluated whether Mygalin could act on other molecular targets such as proteins; for this, we quantified the levels of GSH in *E. coli* following treatment with Mygalin, ciprofloxacin or gentamicin. Treatment with Mygalin and ciprofloxacin produced similar results, reducing the GSH level by 17%. However, GSH was more markedly reduced with gentamicin, with a reduction of approximately 50% (Figure 8A). Due to the low reduction in GSH levels with Mygalin, the influence of this molecule on protein profile expression was investigated (Figure 8B). There were no significant differences in the protein profile between untreated bacteria (1) or bacteria treated with Mygalin (2) or ciprofloxacin (3). However, with gentamicin (4), the protein profile and GSH levels were as previously described. These data suggest that the microbicidal effect of Mygalin involves, in addition to the generation of ROS, other mechanisms already common to antibiotics, since GSH reduction is associated with ROS generation (Belenky et al., 2015).

### Contribution of Mygalin to ROS Generation

**FIGURE 9:**
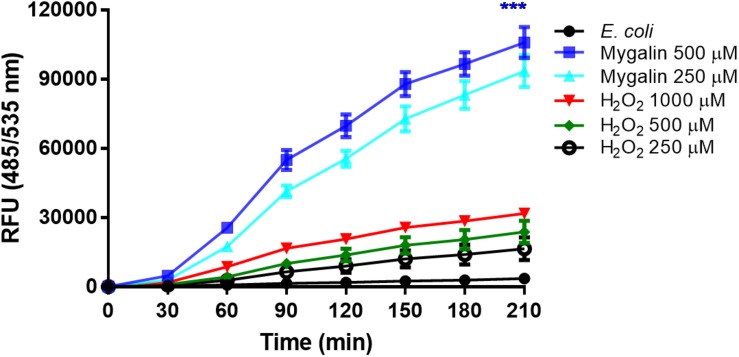
Influence of Mygalin on intracellular ROS generation. The presence of ROS in *E coli* (10^6^ CFU/mL) due to treatment with Mygalin (250 and 500 μM) or H_2_O_2_ (0.25, 0.5, and 1 mM), as a positive control, was studied by measuring the fluorescence of CM-H2DCFDA after 15 min of treatment. RFU = Relative Fluorescence Units. These data represent the mean (±SEM) of three independent experiments (^∗∗∗^*p* < 0.001).

The results of this study showed that Mygalin altered the permeability of the *E. coli* membrane, causing DNA damage. The intrinsic mechanisms used by this molecule to cause *E. coli* death are unknown. One of the common mechanisms of bacterial death caused by antibiotics is the oxidative damage generated by free radicals derived from oxygen, known as ROS (Kohanski et al., 2010). Thus, we investigated whether treatment of *E. coli* with Mygalin induced ROS generation (Figure 9). Bacteria were grown to the exponential phase, and Mygalin (0.25 and 0.5 mM) or H_2_O_2_ control (0.25–1 mM) was added to the cultures. ROS production was monitored for 210 min by reading the fluorescence after adding the fluorophore CM-H2DCFDA (Dong et al., 2015). As shown, the addition of Mygalin to *E. coli* cultures progressively increased the level of ROS between 60 and 210 min of incubation, regardless of the concentration used. These levels were higher than those of the H_2_O_2_ (0.25–1 mM) treatment. These data suggest that one of the possible mechanisms used by Mygalin to promote DNA damage and death of *E. coli* is the generation of intracellular ROS. Similar results were obtained with *E. coli* treated with norfloxacin and ampicillin (Dwyer et al., 2014) using the same fluorophore.

### Interaction of Mygalin With LPS

**FIGURE 10:**
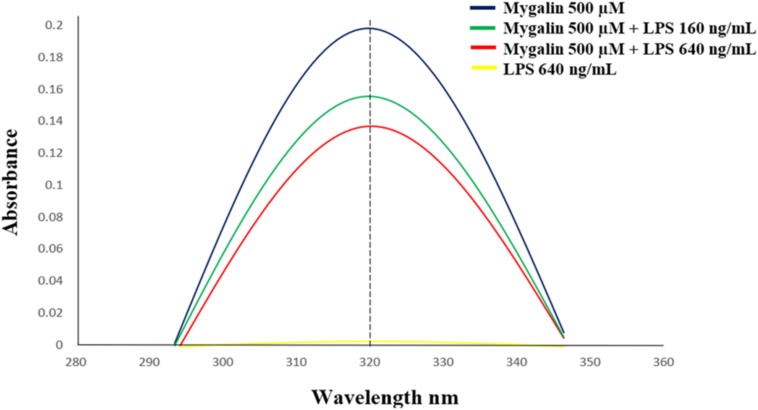
Interaction between Mygalin and LPS. The Mygalin (500 μM)-LPS (160–640 ng/mL) interaction assay was performed after incubation for 1 h at 37°C. The change in absorbance of Mygalin (320 nm) was monitored using a NanoVue Plus^TM^ spectrophotometer according to M&M. A decrease in absorbance intensity in LPS-Mygalin compared to Mygalin alone indicated binding to LPS. The figure is representative of three independent experiments with similar results.

Mygalin exerts microbicidal activity against Gram-negative bacteria only (Pereira et al., 2007), which contain LPS as their main component with major biological activity. It was previously shown that polyamine analogs can neutralize LPS *in vitro* (Miller et al., 2005). Based on this, 0.5 mM Mygalin was incubated with LPS, and the available free Mygalin level was analyzed by spectrophotometry (Figure 10). The incubation of Mygalin with LPS caused a reduction in the absorbance as a function of the increase in the LPS concentration, indicating an interaction between the molecules. Similar results were described with peptides incubated with LPS (Lakshminarayanan et al., 2016; Sinha et al., 2017). Our data suggest that Mygalin may interact with LPS, and this would justify its action only against Gram-negative rather than Gram-positive bacteria.

### Analysis of the Iron Chelating Activity of Mygalin

**FIGURE 11:**
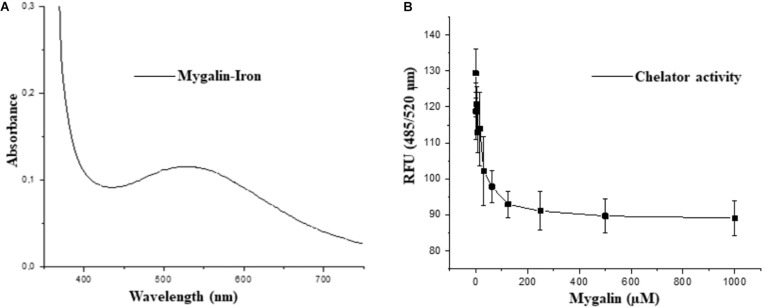
Iron-chelating activity of Mygalin. **(A)** UV-Vis spectrum of 5 mM iron II-Mygalin in water at 25°C after 24 h. Spectrum shows a maximum wavelength at 524 nm. **(B)** The chelating effect of Mygalin (0–1000 μM) on the rate of dihydrorhodamine hydrochloride (DHR) oxidation catalyzed by iron/ascorbate in water at 25°C. The kinetics curve was recorded on a fluorometer with 485/520 nm excitation/emission wavelengths at 25°C for 40 min. The results are the average of quadruplicates. RFU = Relative Fluorescence Units. The figure is representative of three independent experiments with similar results.

The fact that Mygalin is a ROS generating molecule and has structural similarity with siderophore H4-4-LICAM (Raines et al., 2013) suggests that this molecule can function as an iron chelator. Under physiological conditions, labile Fe can generate ROS in the presence of low concentrations of ascorbate. We investigated whether Mygalin could have iron chelating activity because iron is one of the most important metals and is involved in the process of oxidative stress (Halliwell and Gutteridge, 1984). Bacteria were treated with Mygalin (0–1000 μM) followed by the addition of the DHR probe. When the chelator binds to the metal, ROS generation is interrupted, decreasing DHR oxidation. We observed that the addition of increasing concentrations of Mygalin (0–1000 μM) to the system reduced the oxidation of DHR (Figure 11B), which was reflected in the reduction of the spectrum absorption of this molecule due to its association with Fe (Figure 11A).

## Discussion

**FIGURE 12:**
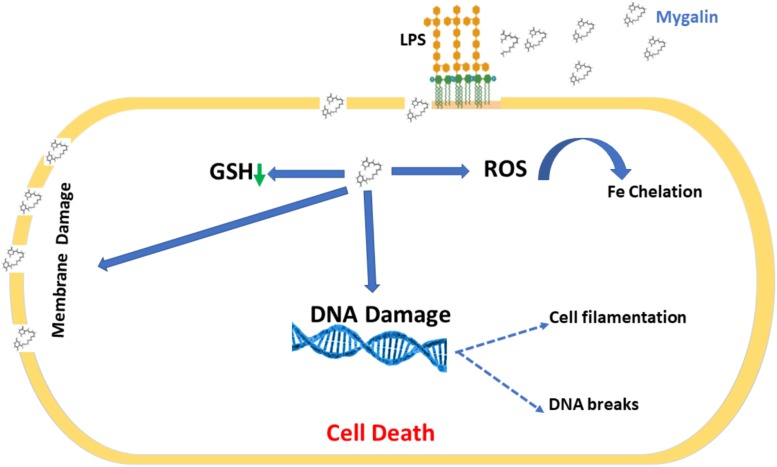
Proposed mechanism for Mygalin action. Mygalin binds to LPS in the outer cell membrane of Gram-negative bacteria; this leads to the rupture of the cell membrane and entry of the drug into the cell, which induces the production of intracellular ROS in the initial hours of cell contact. This leads to the breakdown of bacterial DNA, preventing cell division and inducing bacterial death.

Bacterial drug resistance is a concerning public health problem. The World Health Organization (World Health Organization [WHO], 2001) has encouraged research into the search for new drugs and vaccines to increase the effectiveness of treatment and reduce resistance to antibiotics. It is necessary to find new therapeutic strategies by combining products or defining new target molecules with microbicidal activity. Polyamines participate in the control of virulence genes in microorganisms but have been neglected regarding their bactericidal activity. Several polyamine analogs were constructed, showing significant effector activity alone or in combination with other drugs against tumors (Nair et al., 2007) and bacteria resistant to antibiotics (Blanchet et al., 2016). However, the contribution of analogs to the control of infections is limited. We explored the mechanisms involved in the microbicidal activity of Mygalin using the *E. coli* model *in vitro* as a strategy to evaluate the use of antimicrobial agents. Synthetic Mygalin results from the association of spermidine with gentisic acid, having two acyl groups in its structure. Individually, none of these compounds showed bactericidal activity. This suggests that the microbicidal activity could be attributed to the acyl group present in the structure of Mygalin. This effect was dose-dependent and started after 5 h of contact and was stronger than that of 1 mM H_2_O_2__._ Studies relating the activity and structure of spermine analogs have shown a strong relationship between acyl chain length and antimicrobial potency, suggesting that these analogs could be used to improve the effectiveness of conventional antibiotics (Balakrishna et al., 2006). One of the mechanisms of action of bactericidal drugs is DNA damage due to their fragmentation or protein interaction activities (Kohanski et al., 2007, 2010). The vast majority of the effects of polyamines are associated with their interaction with DNA and RNA (Igarashi and Kashiwagi, 2010). The effect of Mygalin on DNA fragmentation was investigated, and our *in vitro* and *in vivo* data showed that the treatment of genomic or purified DNA from a bacterial culture with Mygalin promoted DNA breakdown. This effect was independent of the pH used in the reactions. In contrast, spermidine did not induce any DNA damage, proving its protective effect (Douki et al., 2000; von Deutsch et al., 2005) and anti-oxidant activity (Tkachenko and Fedotova, 2007; Terui et al., 2018). This shows that both molecules have distinct effects in relation to their action on DNA. The degradation of DNA caused by the treatment of bacteria with Mygalin was independent of DNase since the addition of the enzyme inhibitors did not alter the DNA damage caused by Mygalin. Treatement of *E. coli* with antibiotics promotes oxidative stress and spermidine reduces this effect (Tkachenko et al., 2012), therefore it was analyzed whether this effect could occur when DNA was treated with Mygalin. Our data showed that the addition of spermidine protected DNA samples treated with Mygalin at doses below 0.5 mM. However, this effect did not occur with Mygalin (1 mM) or in the Fenton reaction (positive control), confirming that high concentrations of Mygalin cause irreversible DNA damage. Antimicrobial drugs, as well as antibiotics, have several mechanisms of action (Epand et al., 2016). Factors that influence bacterial viability, such as alteration of cell permeability, release of intracellular components, inactivation of metabolic pathways, inhibition of bacterial growth by chelation of nutrients and metals (Kohanski et al., 2010), were explored to identify the action of Mygalin against *E. coli*. We observed by confocal microscopy that bacteria treated with Mygalin or H_2_O_2_ incorporated DAPI dye, showing that this acylpolyamine can induce DNA damage. In another analysis, the ability of Mygalin to disrupt the cellular membranes of *E. coli* was evidenced since the treated bacteria incorporated PI, similar to that visualized with ampicillin. DNA damage has been described in the treatment of bacteria with antibiotics (Kohanski et al., 2010), toxic metals (Espirito Santo et al., 2011) and antimicrobial peptides (Farkas et al., 2017; Diaz-Roa et al., 2019), which led to bacterial death. Inhibition of DNA synthesis was confirmed by light microscopy (Alfred et al., 2013) during the treatment of *E. coli* with Mygalin and ciprofloxacin, a high bacterial stress-inducing antibiotic (Goswami et al., 2006). This treatment interfered with the cell division cycle of the bacteria and induced the formation of filamentous bacteria. Before *E. coli* divides, several proteins are organized for “Z-ring” assembly, including the FtsZ protein (filament-forming protein), which is essential for cell division and viability of *E. coli* (Addinall et al., 1996). Studies suggest that FtsZ assembly inhibition can be used for the development of drugs against pathogenic bacteria (Wang et al., 2003), as proposed by Rai et al. (2008), who used curcumin to inhibit *Bacillus subtilis* cell division by disrupting the “Z-ring.” Future investigations will be conducted to analyze whether Mygalin also utilizes this mechanism. The esterase enzyme is involved in both ester hydrolysis and lipid peroxidation. An increased esterase level is an indication of drug-induced cell membrane and DNA damage. Treatment of *E. coli* with Mygalin increased fluorescence emission in a dose-dependent manner similar to ampicillin. Therefore, Mygalin can break up the *E. coli* outer membrane, facilitating DNA damage. GSH is essential for redox system regulation and protection against oxidative stress in all cells. The presence of antioxidant mechanisms is necessary to limit the intracellular levels of these compounds. GSH reductase is one of the systems that controls free radical formation and oxidative damage to protect cells against environmental stress (Smirnova and Oktyabrsky, 2005). We observed a slight reduction in GSH reductase levels during the treatment of *E. coli* with Mygalin, without altering the protein profile. This differed from the results of gentamicin treatment, in which there was intense reduction of enzyme and protein profile change in relation to those of the control. Therefore, in addition to Mygalin breaking down the cell membrane and promoting DNA damage, this drug also alters the synthesis of enzymes that participate in the control of oxidative stress in *E. coli*. High levels of free radicals in the intracellular environment can contribute to all of these forms of damage. The presence of intracellular ROS in cells due to treatment with Mygalin was confirmed. There was an intense increase in the level of ROS, which was greater than that observed with 1 μM H_2_O_2_ and proportional to incubation time. Our results indicated that Mygalin promotes DNA damage and the death of *E. coli* through the generation of high intracellular ROS levels, which may contribute to the damage found in previous tests. These considerations support the involvement of ROS in Mygalin-induced *E. coli* death as with certain antibiotics (Dwyer et al., 2014). Previous tests conducted in our laboratory showed that the addition of catalase, thiourea or spermidine to Mygalin-treated *E. coli* cultures recovered bacterial viability by 80% (manuscript in preparation), confirming the importance of the ROS mechanism in *E. coli* death. Experiments with other strains and species of bacteria are being performed in our laboratory to confirm that Mygalin treatment and ROS generation is a common mechanism for killing other bacteria. It was suggested that the microbicidal effect of Mygalin extends to Gram-negative bacteria only (Pereira et al., 2007). LPS is the predominant structural component of the outer membrane of Gram-negative bacteria and is responsible for the death of patients with septic shock in addition to causing intense inflammatory activity (Raetz and Whitfield, 2002). The possible interaction of Mygalin with LPS showed that there was an interaction between these two molecules, partially explaining its action against Gram-negative bacteria. Other drugs, such as curcumin and antimicrobial peptides, have the same ability to interact with LPS, reducing their activity (Lakshminarayanan et al., 2016; Sinha et al., 2017). The presence of chelating metals can interfere with *E. coli* metabolism, leading to its death. The ability of Mygalin to sequester important nutrients for bacterial metabolism was analyzed, and we found a dose-dependent reduction in oxidation of the DRH probe. This means that Mygalin can sequester iron and alter *E. coli* metabolism. The chemical structure of Mygalin resembles that of siderophores. These compounds have the function of solubilizing and capturing iron II for use in bacterial metabolism. A new polyamine, Vulnibactin, which contains siderophore activity, was isolated from *Vibrio vulnificus* by Okujo et al. (1994). This polyamine has two acyl groups similar to Mygalin. This evidence indicates that an increase in intracellular ROS in *E. coli* and reduced iron bioavailability may contribute to Mygalin-induced *E. coli* death. In conclusion, we describe some effector mechanisms of Mygalin, an acylpolyamine, as a new molecule with a microbicidal effect against *E. coli* DH5α. The mechanism of action involves bacterial membrane permeability changes, DNA damage, and ROS generation. In addition, Mygalin chelates iron and binds to LPS. Our data showed that the increase in intracellular ROS associated with iron sequestration and DNA damage in response to Mygalin treatment may be responsible for the death of *E. coli*. Taken together, our data suggest that Mygalin must be explored as a new alternative drug with antimicrobial potential against Gram-negative bacteria and other infectious agents. Based on our results, we propose a mechanism of action of Mygalin, as shown in Figure 12. Studies are underway to confirm these effects and the consequences of this oxidative stress on other bacterial groups and biofilm formation. The most interesting point of this study is that this molecule does not promote cytotoxicity in eukaryotic cells and may interfere with the innate immune response, another aspect that is being explored.

## Data Availability Statement

The raw data supporting the conclusions of this article will be made available by the authors, without undue reservation, to any qualified researcher.

## Author Contributions

AE-C, PS, and MB carried out the study design, conducted the study, and wrote the manuscript. EM and HV helped with the experiments.

## Conflict of Interest

The authors declare that the research was conducted in the absence of any commercial or financial relationships that could be construed as a potential conflict of interest.
